# From the President’s Desk

**DOI:** 10.4102/safp.v63i1.5321

**Published:** 2021-05-12

**Authors:** Robert Mash

**Affiliations:** 1 Division of Family Medicine and Primary Care, Faculty of Medicine and Health Sciences, Stellenbosch University, Cape Town, South Africa

Our National Family Practitioners Conference will go ahead 13–14 August 2021 (https://saafp2021.org.za). I ask all of you to diarise the dates and plan to participate! The conference will be fully virtual, which will enable many more people to attend. You will have no costs for travel or accommodation, and registration fees will also be lower than usual as there is no venue to hire. We hope that many people from the African region will also join us. We have signed a contract with Scatterlings, a conference organiser, who will provide a state-of-the-art virtual platform and help organise the conference.

The theme of the conference is family medicine in the era of coronavirus disease 2019 (COVID-19), and plenary speakers will address a host of clinical and service delivery questions related to the theme. We plan a mix of high-quality international and national speakers as the virtual platform gives us a global reach. We will also have a variety of breakaway practical workshops, thematic seminars and research presentations. The programme is being organised by Prof. Indiran Govender with a team from the University of Pretoria and the Academy Executive. We will shortly be communicating with more information on the programme and how to register.

Over the next few weeks, we also intend to disseminate a survey to our members on your needs for continuing professional development. We would like to identify and prioritise your needs and to develop on-line short courses to address them. Prof. Selma Smith will assist the Academy in developing these short courses.

On our website, you will also see that there is now a category for medical student membership (https://saafp.org/about-us-2/membership-2/). We would like to encourage medical students interested in family medicine and primary healthcare to join the Academy and explore the discipline as a future career pathway. We hope that Faculties of Medicine and Health Sciences and their Departments of Family Medicine will assist students to establish special interest groups and to facilitate these groups to join the Academy.

Over the last few weeks, I have been visiting rural district hospitals in the Western Cape, where we are training registrars in family medicine. One of the big changes this year is the introduction of second year interns in family medicine and primary care for a 6-month rotation. Districts have differed considerably in how they have organised these rotations. One district decided to not have any interns, another has placed them all at the district hospitals with their associated primary care platforms, while another has them rotating between a district hospital, a community health centre and a regional hospital emergency department. The feedback at all these district hospitals has been that interns are appreciated as a valuable addition to the team and are making a difference, while interns say they are enjoying the opportunities to take clinical responsibility and obtain valuable practical experience.

We are building a pipeline for the discipline from medical students to interns, registrars and eventually family physicians. We hope that these pre-service and in-service experiences will attract more junior doctors to specialise in family medicine. We also advocate with the public and private sectors to create more posts and opportunities for family physicians.

During my visit to the district hospitals, the number of COVID-19 patients was few, and the second wave was over. However, most hospitals were maintaining their COVID-19 wards in anticipation of a third wave, and most of the staff were slowly receiving their vaccinations. It seems that COVID-19 will continue to dominate our minds for the rest of 2021. In the Western Cape, the next phase must roll out vaccinations to 900,000 older people and people with co-morbidities. In the midst of this, we must find ways of maintaining and restoring services for people with other health problems and conditions. Healthcare workers are also battered by the pandemic with many traumatised at work and bereaved at home by the experience. We must find ways to debrief and support each other.

